# *In situ* Transmission Electron Microscopy observation of Ag nanocrystal evolution by surfactant free electron-driven synthesis

**DOI:** 10.1038/srep21498

**Published:** 2016-03-16

**Authors:** Elson Longo, Waldir Avansi, Jefferson Bettini, Juan Andrés, Lourdes Gracia

**Affiliations:** 1 Institute of Chemistry, UNESP−Universidade Estadual Paulista, R. Francisco Degni, 55, Araraquara 14800-900, Brazil; 2 Department of Physics, UFSCar− Universidade Federal de São Carlos, Rod. Washington Luis, km 235, Sao Carlos 13565-905, Brazil; 3 Brazilian Nanotechnology National Laboratory (LNNano), R. Guiuseppe Maximo Scolfaro 10000, Campinas, 13083-970, Brazil; 4 Departament de Química Física i Analítica, UJI−Universitat Jaume I, Av. de Vicent Sos Baynat, s/n, Castelló de la Plana 12071, Spain

## Abstract

The study of the interaction of electron irradiation with matter and the response of the material to the passage of electrons is a very challenging problem. However, the growth mechanism observed during nanostructural evolution appears to be a broad and promising scientific field in nanotechnology. We report the *in situ* TEM study of nanostructural evolution of electron-driven silver (Ag) nanocrystals through an additive-free synthetic procedure. Observations revealed the direct effect of the electron beam on the morphological evolution of Ag nanocrystals through different mechanisms, such as mass transport, site-selective coalescence, and an appropriate structural configuration after coalescence leading to a more stable configuration. A fundamental understanding of the growth and formation mechanisms of Ag nanocrystals, which interact with the electron beam, is essential to improve the nanocrystal shape-control mechanisms as well as the future design and study of nanomaterials.

The fundamental process for nanomaterial formation has received increasing attention in recent years. Specifically*, in situ* electron microscopy appears to be a powerful and promising technique for the direct study of crystal nucleation and growth at the nanoscale, which simultaneously provides both morphological and crystallographic information^1^,^2^,^3^,^4^,^5^,^6^,^7^,^8^,^9^,^10^,^11^,^12^. However, knowledge about nucleation, crystal growth mechanisms, and the direct influence that the electron beam can have in these processes presents a scientific barrier in the research field of nanotechnology^1^,^2^,^3^,^4^,^5^,^6^,^7^,^8^,^9^,^10^,^11^,^12^,^13^,^14^,^15^.

In solid state, the function value that can be obtained from the interaction between electrons and matter is highly dependent on the degree of organization and complexity of the matter that will interact with the electrons. Recently, our research group reported the unprecedented real-time *in situ* observation of the growth process of Ag metallic filaments from the unstable α-Ag_2_WO_4_ crystal matrix when submitting the crystal to electron irradiation from the transmission electron microscopy (TEM)^16^. This is a remarkable result leading to the synthesis of Ag/α-Ag_2_WO_4_ nanostructures; furthermore, the mechanism associated with the initial events of the Ag nucleation process was studied in detail^17^. This behavior can be associated to an excitation inside the nanoparticles, which might significantly change the interaction forces between them, and can be considered a clear example of the formation of compact hybrid semiconductor-metal nanoparticles. All these effects should be taken into account in order to understand the process characteristics in TEM. Owing to its potential applications in optical fibers, photocatalytic materials, and sensors, α-Ag_2_WO_4_ has attracted considerable research and development attention^18^,^19^,^20^,^21^,^22^,^23^,^24^,^25^,^26^,^27^,^28^,^29^,^30^,^31^. By exposure to the electron beam of an electron microscope, the α-Ag_2_WO_4_ undergoes *in situ* nucleation of Ag filaments on the crystal surface^16^,^17^,^32^, and the presence of these Ag nanoparticles leads to unique optical^33^ and microbial properties^34^.

Actually, Ag nanostructures are emerging materials with innovative and enhanced properties, such as catalytic, antibacterial, and optical properties^35^,^36^,^37^,^38^. Much of the research in this area is focused on the application of a wide range of experimental techniques and theoretical studies to provide a basic understanding of the growth mechanism, electronic structure, and physical/chemical properties of the Ag nanostructures^35^,^36^,^39^,^40^,^41^,^42^,^43^,^44^. In this communication, we describe an interesting process: the formation and growth of Ag nanocrystals (NCs) from Ag filaments^16^ occurred spontaneously over time after the irradiation process on the α-AgWO_4_ crystal. The growth process was also induced by electron exposition. Based on this knowledge, the main aim of the present study was to collect essential information on the growth process of Ag nanoparticles formed from Ag filaments on the α-Ag_2_WO_4_ nanostructures when exposed to TEM electron beam irradiation.

## Results

**Figure 1:**
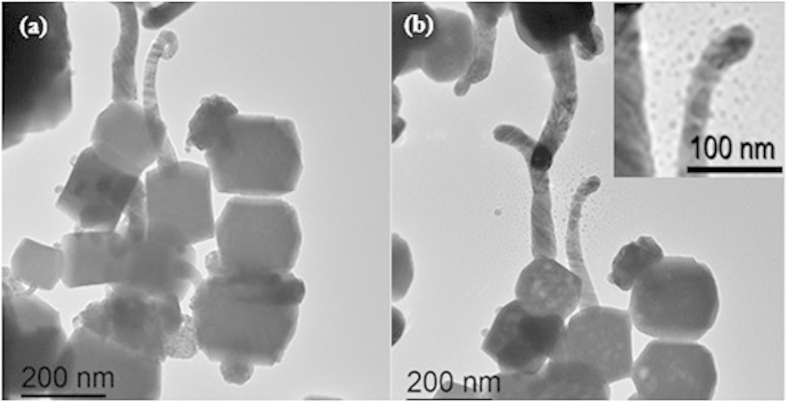
TEM images of α-Ag_2_WO_4_ NCs: (**a**) α-Ag_2_WO_4_ after electron beam exposure and (**b**) α-Ag_2_WO_4_ after 148 min under electron beam exposure; (the inset shows a magnified image of the formed Ag nanoparticles).

Figure 1 presents a typical image of α-Ag_2_WO_4_ NCs after the growth of Ag filaments stimulated by the electron beam irradiation on the α-Ag_2_WO_4_ surface. Our group, based on a combination of theoretical and experimental results, reported that the nucleation and growth of Ag filaments on the α-Ag_2_WO_4_ resulted from the order/disorder effects generated in the crystal when electron irradiation induced a structural and electronic rearrangement^17^,^33^.

Nevertheless, in the present study, an analysis performed on Ag filaments during the experiment presents new phenomena. Figure 1 shows that the continuous exposure of the electron beam leads to a decrease in the dimensions of the Ag filament accompanied by the appearance of new nanostructures around the Ag filament (inset of Fig. 1b). Upon exposure to the TEM electron beam, the filaments become unstable and Ag atoms are deposited on the carbon film, leading to the formation of Ag nanoparticles between 2 and 15 nm after a period of exposure, Fig. 1b. In other words, there is a transformation from a quantum cluster to a metallic particle with the continuous growth of the nanoparticles driven only by electron beam exposure.

The nucleation and growth of Ag NCs are initiated by irradiating the α-Ag_2_WO_4_ with the electron beam. A burst of nucleation is observed when the sample is focused for imaging, followed by a continuous appearance of new nanoparticles. Although nanoparticle coalescence events occur in the early stage of growth, most nanoparticles eventually develop into isotropic nanostructures *via* the attachment of monomeric species.

**Figure 2:**
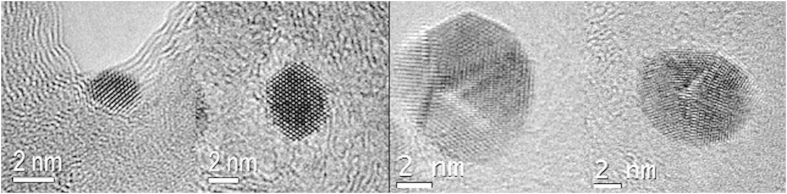
Representative TEM images of Ag NCs formed under electron beam irradiation.

Some representative images of individual Ag NCs obtained from the region around the electron-driven Ag filament are illustrated in Fig. 2. It is possible to identify a single crystalline nature and some morphological characteristics of the Ag NCs formed during these experiments, with dimensions between 2 and 15 nm. The formation of these NCs may be related to the mass transfer along the carbon film through the particle motion induced by the electron beam exposure and followed by a reorganization process. Indeed, with the increased dimensions, a more faceted NC with a more stable energetic configuration can be observed.

**Figure 3:**
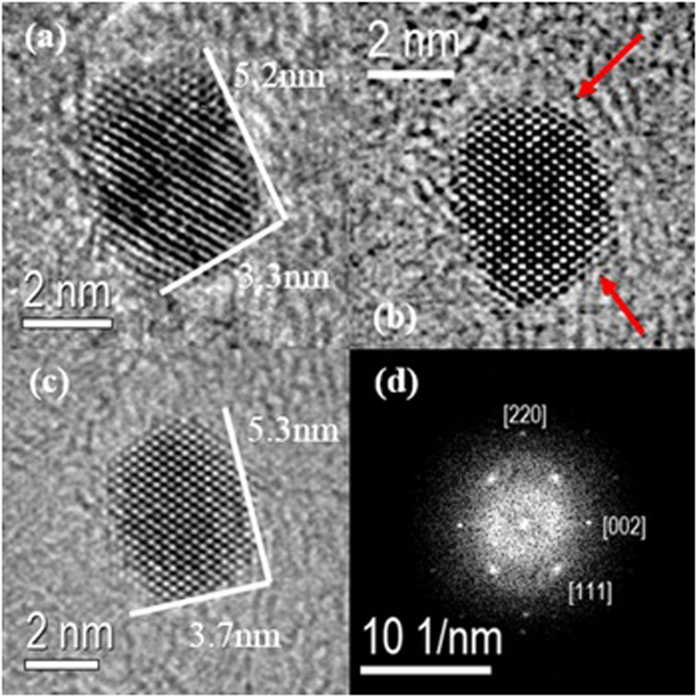
(**a–c**) HRTEM image of NC growth under electron beam irradiation from Movie S3. (**d**) Fourier Transform (FT) of the individual NC shown in (**c**).

We analyzed the evolution in shape of an Ag NC by tracking the propagation of different facets during a temporal sequence of high-resolution TEM (HRTEM) images, as shown in Fig. 3(a–c). The Fourier transform (FT) of the illustrated nanocrystal confirms that the NC has the Ag cubic crystallographic structure and a single crystalline nature, Fig. 3d. Despite the evident deficiency of Ag sources and their relatively low mobility compared to liquid cell experiments^2^,^3^,^11^, the time-resolved figure clearly presents the growth of an NC that leads to a well-faceted shape with the motion of atoms at the two well-resolved {111} facets, as indicated by the red arrows.

**Figure 4:**
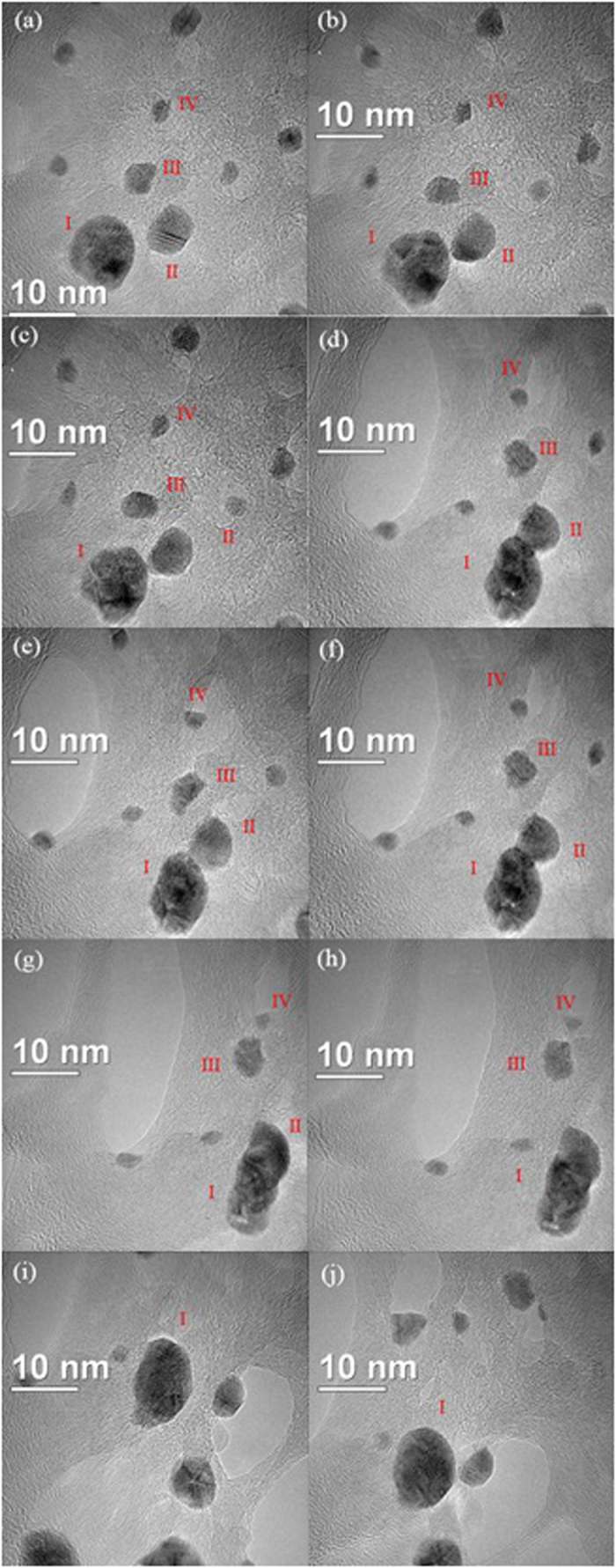
(**a**–**j)** Sequence of *in situ* TEM images from Movies S1 and S2 showing an example of the attachment and rearrangement of the NCs after coalescence, and consumption of the attached NC.

In addition to the formation of Ag NCs by a mass transfer process, the *in situ* time-resolved TEM images in Fig. 4 (see Movie S1 and S2) revealed that the NCs also grow as a result of two monomer or primary nanoparticle additions from coalescence events. This process is suggested in Fig. 4 by the presence of an isotropic nanocrystal (denoted as I) surrounded by several other NCs. Indeed, under continuous electron irradiation, Fig. 4(a–h) shows that NC II is trapped and then fused across a mismatched interface after approximately 725 s. At t = 0 s, it was possible to observe a distance of approximately 1.5 nm between the NCs I and II. Then, with an increased duration of electron irradiation exposure, this distance decreases to the point of attachment of both NCs (Fig. 4a–d).

**Figure 5:**
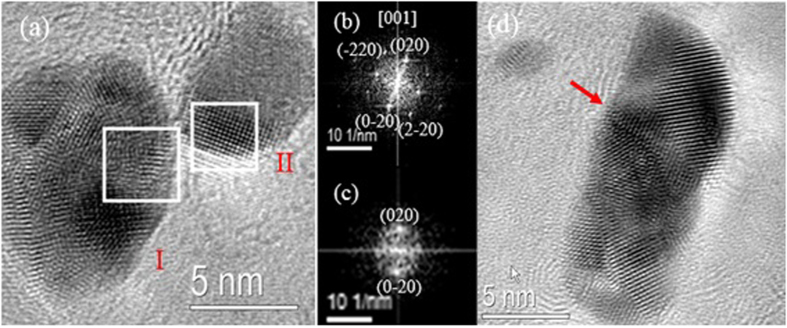
(**a**) HRTEM image from Movie S1 showing the attachment across a mismatched interface; (**b**,**c**) the Fourier transform (FT) of the illustrated region for each NC, (**d**) HRTEM image after the fusion between NCs I and II.

It is interesting to note that the NC I also undergoes a structure rearrangement with the approximation and coalescence of NC II (as shown in Fig. 4a–d). *In situ* time-resolved HRTEM images in Fig. 5 also show that at the time of attachment, the grain boundaries migrated towards the adjacent particle, leading to the growth and consequently a rearrangement of nanoparticle, a process that was completed after approximately 765 s (Fig. 4a–i). After contact, the neck formation between the NCs could be observed, which occurs via a coalescence process similar to the ones observed by *in situ* TEM studies related to other materials using liquid cells^2^,^3^,^11^. Indeed, the HRTEM analysis (Fig. 5a–c) shows that NCs I and II have a misorientation in the interface, where the arrows on Fig. 5d also revealed the defect formation during the coalescence and rearrangement processes. This behavior was expected once the crystal growth, which involves an imperfect attachment between two small misoriented NCs, led to the presence of defects in the NCs^45^,^46^. In addition to the behavior observed for NCs I and II, an interesting event was also detected for NCs III and IV, where their shape varied with time, as shown by the analysis of the images in Fig. 4.

**Figure 6:**
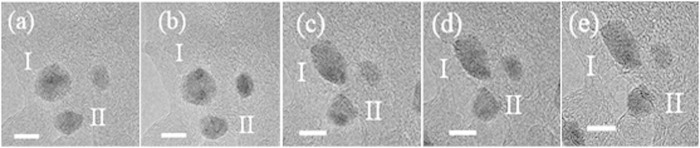
Sequence of *in situ* TEM images from Movie S3 (region A of Fig. S1) showing an example of rearrangement without NC attachment: (**a**) t = 0 s; (**b**) t = 30 s; (**c**) t = 223 s; (**d**) t = 255 s and (**e**) t = 345 s. The scale bar is equal to 5 nm.

Figure 6 presents the time-resolved TEM images of the trajectories and rearrangement of three illustrated NCs under electron irradiation. Initially, one nanocrystal (denoted as II) starts to move in the direction of NC I; when t = 0 s, the distance (d) is 3.5 nm; after NC II approached to within 1.5 nm, the movement reversed, leading to a distance increase between the two crystals to 2.7 nm (Fig. 6e). Indeed, after t = 223 s, the NC I presents an elongated configuration in proximity to NC II. This behavior provided evidence for more complicated electrostatic interactions, similar to those that have been reported to explain the iron oxides motion^2^, which were attributed to the presence of cation-cation and anion-anion repulsion when the lattices were mismatched, or to the presence of defects in the NC surface^2^.

**Figure 7:**
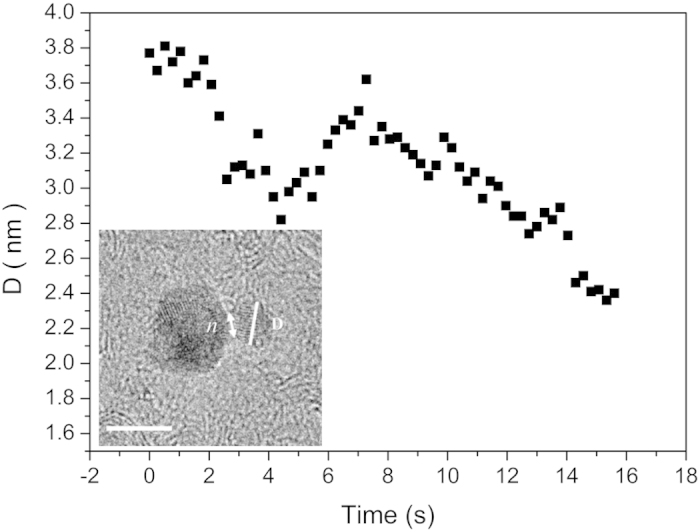
Decrease of D with time for NC III from Movie S3. TEM image (inset) illustrating the attachment between NCs III and IV with a neck formation (see Fig. S1). The scale bar is equal to 5 nm.

The sequence for the other region of Movie S3 (region B illustrated in Fig. S1) presents details about the behavior related to the motion of a small nanoparticle (denoted as III) with a velocity of around 0.005 nm s^–1^, which was completely consumed by the bigger one (denoted as IV). This mechanism, *i.e.*, the dissolution of small particles in proximity of bigger ones is driven by the Ostwald ripening mechanism^4^,^6^,^47^. After the attachment, the dependency of diameter size (D) with time for NC III during its consumption can be observed in Fig. 7. The analysis of Fig. 7 shows an interesting and different dissolution kinetics than those observed in other studies, appearing to occur in cycles^3^,^6^,^11^. After attached, a neck formation occurred between NCs III and IV with *n* = 1.6 nm, Fig. 7 (inset). With t = 4.5 s, D stops decreasing and this fact was repeated at t = 9.4 and 12.8 s. Indeed, at t = 4.5 s a decrease in neck size was observed (*n* = 1.4 nm), and identified in each end of the cycle (Fig. S1). The end of the dissolution was followed by an increase in D, suggesting the electron-driven rearrangement of the structure, where the value of *n* remained constant. Moreover, for t = 9.4 and 12.8 s, the observed *n* is equal to 1.0 nm. Despite the fact that the 3D shape dependence could not be analyzed, it is clear that the decrease in contact area between nanoparticles, observed by the decrease in neck dimensions, is different from that observed in other *in situ* TEM studies^11^.

Recently, first-principles calculations were carried out by our group using the density functional theory (DFT) to reveal the Wulff’s construction of the optimized Ag crystal and the different morphologies assuming different surface energy ratios^48^. Indeed, the analysis reveled that the calculated energy surface values generate a morphology very similar to that of the synthesized Ag NCs, observed by TEM images^48^.

## Discussion

In addition to the formation of Ag NCs by a mass transfer process, the crystallization process of the NCs was observed in multiple stages as follows. From Fig. 4a–c: interaction between the flanked NCs; Fig. 4d–f: mutual alignment between the NCs, followed by different synergic formation processes to produce mesocrystals *via* oriented attachment; and Fig. 4g,h: by fusing together, the NCs face elimination and reduction of surface energy, followed by a structural arrangement with increased symmetry. Additionally, in an intermediate stage shown in Fig. 5d, the NC I clearly presented an unstable configuration during the growth process, with the breaking of the symmetric morphology leading to an initial detachment process of the other three nanocrystals; this process was stopped due to the structure rearrangement and complete consumption of the NC II.

The synthesis of Ag NCs has been extensively studied, and Stamplescoskie *et al.* have investigated whether an electron magnetic field can direct the Ag crystal growth, where the morphology of the synthesized NCs is related to the excitation wavelength provided by the laser^49^. With regard to the synthesis using an electron beam, studies on metal nanoparticle synthesis and growth have been performed on liquid cell TEM using an electron beam source as the reducing agent to reduce metal precursors in the growth solution^9^,^10^. According to Woehl *et al.* the electron beam has important effects on the growth mechanism of Ag NC synthesis in solution, producing bubbles in liquids, solvated ions or electrons, and rupture in the liquid film^9^,^10^. In this sense, it is well known that the investigation of reactions and crystal growth mechanisms under electron beam irradiation is very complicated and requires new and deeper investigations^9^,^10^. In fact, our results bring a new insight about the effects of the electron beam in crystal growth studies. In this case, the observed effects are completely free of some factors commonly present in liquid cell TEM, such as additional agents (organic or inorganic ions), activity dependence in solution media, and/or the effect of the electron beam in solution, which can interfere with the process^9^,^10^.

A careful analysis of Figs 4 and 5 clearly also reveals that the NCs move to an appropriate configuration to attach and form a neck at the initial stage of the coalescence. This process has also been observed in previous studies related to crystal growth mechanisms^2^,^3^,^11^. The attachment of the NC pairs occurs in the {010} crystallographic direction (see Fig. 5c) followed by a neck growth with configuration changes and a migration process of atoms due to surface diffusion, as observed in Fig. 4 50. However, from time-resolved TEM analysis (Figs 4, 5, 6), the size dependence between the two NCs involved in the growth process cannot be analyzed due to their 3D shape. On the other hand, under the present experimental conditions, as noted above, the sequence of images presented in Fig. 4 also reveal that during and after the process of coalescence, the nanostructures gradually reorganize and develop faceted surfaces that produce isometric morphologies.

The above analysis clearly illustrates that the NCs under electron beam irradiation explore several configurations before movement, attachment and growth (Figs 4, 5, 6). This behavior is possible because the observed NCs tend to aggregate into clusters to interact in close proximity, suggesting the existence of an attractive force that leads to the coalescence of the NCs^2^,^51^. From the analysis of the images presented in Figs 4 and 6, another interesting behavior can be observed when the particles tend to remain separated by nanometer-scale distances or even farther apart. Batson *et al.*^51^ observed the presence of the attractive and repulsive forces during the plasmonic response to the electron beam passage; consequently, the forces are dependent on the parameters of the electron beam and not only related only to crystal configuration.

Indeed, after an initial coalescence stage, it is expected an increase in *n* with decrease NCs dimensions, due to atom migration towards the neck region^11^. The dissolution rate and its dependency on size also appears similar between different cycles, in contrast with the dependency observed by iron oxide nanoparticle growth^11^. Recently, Sun *et al.* demonstrated the variation in shape of Ag NCs dominated by surface diffusion^8^. This mechanism also appeared to occur in this study, with an oscillatory behavior of the NC III dissolution kinetics after attachment, leading to a migration toward bigger nanoparticles by atoms surface diffusion. This fact can be related to an antibonding configuration between NCs after surface diffusion, which is followed by a structural rearrangement to an appropriate configuration in order to continue the dissolution of small NCs, resulting in oscillatory consuming characteristics. A similar process was reported by Xin *et al.* who observed an oscillatory growth of Bi nanoparticles at an elevated temperature, attributed to the presence of a mass transport zone around the nanoparticles^47^. Additionally, electromagnetic radiation can interact strongly with metallic nanoparticles through localized surface plasmon resonance^51^. Therefore, our results demonstrate the possible presence of bonding and antibonding configurations between nanoparticles, corresponding to the interaction of individual particle plasmons, and the different surface configurations during the electron-driven growth process.

One of the most important aspects in the crystal growth studies in solution has been attributed to the nanoparticle motion, which could be strongly related to several factors, such as Brownian movement, chemical environment, liquid flow, etc^4^. Indeed, the presented results clarify the effect of the electron beam on the growth, motion, and rearrangement of Ag NCs. The mechanism of the Ag NC growth process took place *via* site-selective coalescence, structural rearrangement during and after coalescence or dissolution, and an evolution of the growth mechanism with surface faceting leading to a more stable configuration. The coalescence observed in this study proceeded with contacts that joined identical or mirroring {010} planes, similar to processes observed in other materials^2^,^6^,^45^,^46^. The NCs observed in Figs 4 and 5 exemplify this process, which results in a perfectly aligned crystal with a single crystallographic domain, as shown in the FT pattern in Fig. 5d. However, it appears that, under the present experimental conditions, structural rearrangements of the NCs occurred not only *via* surface rearrangements, but also *via* reorganization of the complete NC interior, maintaining their crystalline nature.

In summary, we report a complex process involving the formation and subsequent growth of Ag NCs through an unexpected additive-free *in situ* fabrication process and crystal growth mechanism under electron beam irradiation, from the formation of compact hybrid semiconductor-metal nanoparticles. These processes were carefully investigated and our results clarified the influence of electron beam irradiation on metallic nanoparticles and emphasize the importance of a systematic analysis and their effects for *in situ* studies to confirm the underlying mechanisms for nucleation and growth.

## Methods

### Synthesis

The synthesis route employed to obtain the α-Ag_2_WO_4_ nanostructures was described in great detail by Longo *et al.*^16^. Initially, the precursors were prepared by separately dissolving 1 × 10^–3^ mol of tungstate sodium (Na_2_WO_4_·2H_2_O) and 1 × 10^–3^ mol of silver nitrate (AgNO_3_) in 50 mL of deionized water. Then, the α-Ag_2_WO_4_ nanostructures were obtained at 90 °C by injecting the as-prepared precursors into hot aqueous solutions. The as-obtained suspensions were washed several times with deionized water to remove any remaining sodium ions.

### Characterization

*In situ* TEM analysis was performed on a Jeol JEM 2100F with a field emission gun (FEG) operating at 200 kV. The samples were prepared by a simple colloid dropping of the as-prepared α-Ag_2_WO_4_ on amorphous carbon film supported on cupper (Cu) grids. The electron dose used during all process was kept between 150 and 250 e/A^2^.s for the formation and nucleation process, and the movie was acquired in similar doses.

## Additional Information

**How to cite this article**: Longo, E. *et al.* *In situ* Transmission Electron Microscopy observation of Ag nanocrystal evolution by surfactant free electron-driven synthesis. *Sci. Rep.* **6**, 21498; doi: 10.1038/srep21498 (2016).

## Supplementary Material

Supplementary Information

Supplementary Movie S1

Supplementary Movie S2

Supplementary Movie S3
