# Supplementary material for: In situ Transmission Electron Microscopy observation of Ag nanocrystal evolution by surfactant free electron-driven synthesis
Source: Sci Rep. 2016 Mar 16;6:21498. doi: 10.1038/srep21498 (PMC4793220; doi:10.1038/srep21498)
Supplement: Supplementary Information [file srep21498-s1.pdf]

Supplementary Information for

In situ Transmission Electron Microscopy observation of Ag nanocrystal evolution by surfactant free electron-driven synthesis.

Authors: Elson Longo,[1] Waldir Avansi Jr.[2], Jefferson Bettini[3], Juan Andrés[4] and L. Gracia[4]

Affiliations:
[1] Institute of Chemistry, UNESP−Universidade Estadual Paulista, R. Francisco Degni, 55, Araraquara 14800-900, Brazil

[2] Department of Physics, UFSCar− Universidade Federal de São Carlos, Rod. Washington Luis, km 235, Sao Carlos 13565-905, Brazil

[3] Brazilian Nanotechnology National Laboratory (LNNano), R. Guiuseppe Maximo Scolfaro 10000, Campinas, 13083-970, Brazil

[4] Departament de Química Física i Analítica, UJI−Universitat Jaume I, Av. de Vicent Sos Baynat, s/n, Castelló de la Plana 12071, Spain

*Correspondence to: elson@iq.unesp.br

1. Supporting Movie Legends

Movie S1. This movie shows the sequence of *in situ* TEM images showing an example of appromation and attachment of the Nanocrystal.

Movie S2. This movie shows the sequence of *in situ* TEM images showing the rearrangement of the Nanocrystal.

Movie S3. This movie presents *in situ* TEM images showing the trajectories and rearrangement of lacked Nanocrystal.

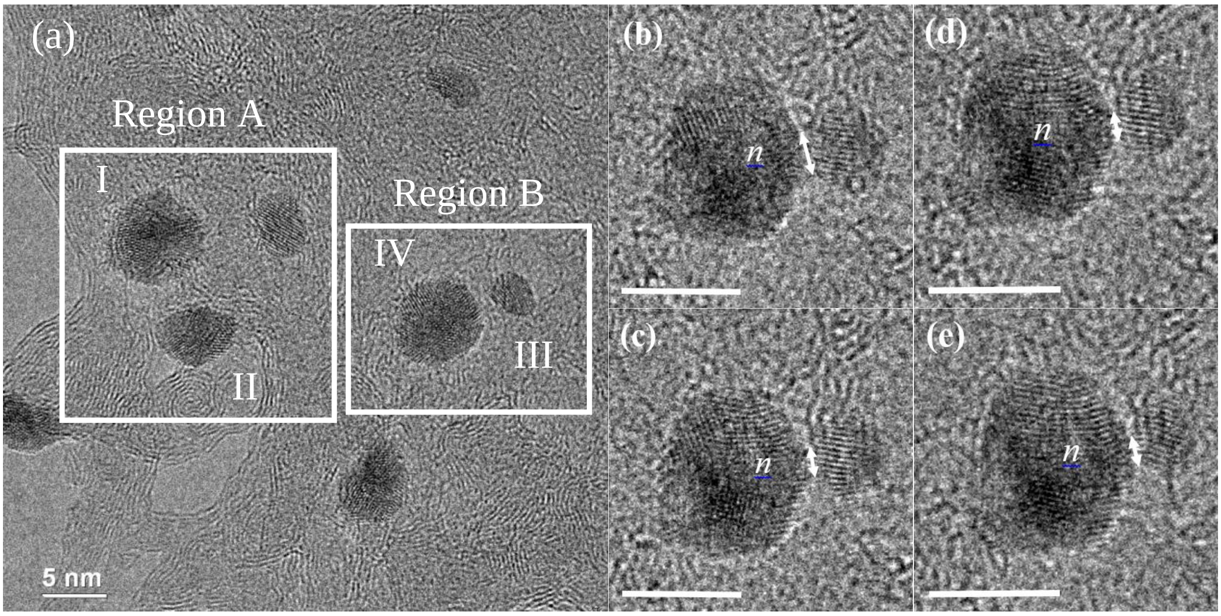

Figure S1: Representative in situ TEM images from Movie S3. (b)-(e) Sequence of an expanded view of region B illustrating the attachment between NC III and IV with formation of neck; (b) t = 4.5s; (c) t=7.4s; (d) t = 9.4s and (e) 12.8s. Scale bars equal to 5 nm.
